# Development and Validation of Prediction Models for Perceived and Unmet Mental Health Needs in the Canadian General Population: Model-Based Synthetic Estimation Study

**DOI:** 10.2196/66056

**Published:** 2025-02-19

**Authors:** Jianli Wang, Heather Orpana, André Carrington, George Kephart, Helen-Maria Vasiliadis, Benjamin Leikin

**Affiliations:** 1 Department of Community Health and Epidemiology, Faculty of Medicine, Dalhousie University, 5790 University Avenue, Halifax, NS, B3H 1V7, Canada, 1 9024943860; 2 Department of Psychiatry, Faculty of Medicine, Dalhousie University, Halifax, NS, Canada; 3 Centre for Surveillance and Applied Research, Public Health Agency of Canada, Ottawa, ON, Canada; 4 School of Psychology, University of Ottawa, Ottawa, ON, Canada; 5 Clinical Epidemiology Program, Ottawa Hospital Research Institute, Ottawa, ON, Canada; 6 School of Health Administration, Faculty of Health, Dalhousie University, Halifax, NS, Canada; 7 Department of Community Health Sciences, Faculty of Medicine and Health, Université de Sherbrooke, Sherbrooke, QC, Canada; 8 Community Health and Wellness Branch, Ottawa Public Health, Ottawa, ON, Canada

**Keywords:** population risk prediction, development, validation, perceived mental health need, unmet mental health need

## Abstract

**Background:**

Research has shown that perceptions of a mental health need are closely associated with service demands and are an important dimension in needs assessment. Perceived and unmet mental health needs are important factors in the decision-making process regarding mental health services planning and resources allocation. However, few prediction tools are available to be used by policy and decision makers to forecast perceived and unmet mental health needs at the population level.

**Objective:**

We aim to develop prediction models to forecast perceived and unmet mental health needs at the provincial and health regional levels in Canada.

**Methods:**

Data from 2018, 2019, and 2020 Canadian Community Health Survey and Canadian Urban Environment were used (n=65,000 each year). Perceived and unmet mental health needs were measured by the Perceived Needs for Care Questionnaire. Using the 2018 dataset, we developed the prediction models through the application of regression synthetic estimation for the Atlantic, Central, and Western regions. The models were validated in the 2019 and 2020 datasets at the provincial level and in 10 randomly selected health regions by comparing the observed and predicted proportions of the outcomes.

**Results:**

In 2018, a total of 17.82% of the participants reported perceived mental health need and 3.81% reported unmet mental health need. The proportions were similar in 2019 (18.04% and 3.91%) and in 2020 (18.1% and 3.92%). Sex, age, self-reported mental health, physician diagnosed mood and anxiety disorders, self-reported life stress and life satisfaction were the predictors in the 3 regional models. The individual based models had good discriminative power with C statistics over 0.83 and good calibration. Applying the synthetic models in 2019 and 2020 data, the models had the best performance in Ontario, Quebec, and British Columbia; the absolute differences between observed and predicted proportions were less than 1%. The absolute differences between the predicted and observed proportion of perceived mental health needs in Newfoundland and Labrador (−4.16% in 2020) and Prince Edward Island (4.58% in 2019) were larger than those in other provinces. When applying the models in the 10 selected health regions, the models calibrated well in the health regions in Ontario and in Quebec; the absolute differences in perceived mental health needs ranged from 0.23% to 2.34%.

**Conclusions:**

Predicting perceived and unmet mental health at the population level is feasible. There are common factors that contribute to perceived and unmet mental health needs across regions, at different magnitudes, due to different population characteristics. Therefore, predicting perceived and unmet mental health needs should be region specific. The performance of the models at the provincial and health regional levels may be affected by population size.

## Introduction

Mental disorders are prevalent and have a considerable impact on the people who experience them, as well as families, communities and society as a whole. To enhance the provision of timely and appropriate mental health services for people who are in need, effective health services planning should be adaptable and responsive to the changing needs and emerging opportunities. Research has shown that many people living with a mental disorder do not receive the services due to the barriers of availability, accessibility, and acceptability [1]. On the other hand, many people with symptoms of mental disorders that do not meet clinical criteria may also benefit from intervention and these people may actively seek treatment to prevent the symptoms from escalating to the level of a disorder [2]. These phenomena reflect different types of mental health care needs and the extent to which the mental health needs are being met.

Mental health need is difficult to define. Bradshaw [3] proposed six need types: (1) normative (presence of a mental disorder diagnosis), (2) felt (subjective perception of a mental health problem), (3) expressed (demand for mental health service), (4) comparative (population inequities in mental health), (5) medical (treatable disease), and (6) social (restoring quality of life). There is evidence to suggest that perceptions of a mental health need (felt need) are closely associated with service demands [4] and are an important dimension in needs assessment [5]. Data-driven evidence about perceived and unmet mental health needs at provincial, state and regional, or county levels can be very helpful in building the business case of demand [6]. However, population health data about perceived and unmet mental health needs have not been well adopted for this purpose [7].

Unmet health need is “the absence of sufficient or appropriate care and services.” [8] These needs are commonly assessed as being for information, medication, counseling or therapy, or another type of help. A need is considered to be fully met when a person receives help meeting all their expectations. In other cases, the help may only partly fulfill the need, or no support is provided at all and the need is unmet [9]. Based on 2012 Canadian Community Health Survey (CCHS)–mental health, 17.3% of Canadians aged 15 years and older reported having mental health needs in the past year, and about one-third of those with a mental health care need reported that it was unmet [9,10]. Addressing unmet needs is important, as people with untreated anxiety or depression are at higher risk of experiencing poor outcomes including persistence of symptoms, delayed recovery, poor personal and occupational functioning, and recurrence of these problems [11]. Unmet health care need has been identified to be an important indicator of health care access within various health care systems [12,13], and is a form of inefficiency that has economic implications, according to Mental Health Commission of Canada [14]. Both perceived and unmet mental health needs constitute the central part of demand which is a critical element in the process of health resources allocation.

Decision makers and health service planners have detailed information about existing mental health resources including available infrastructure, services and workforce at the provincial and regional levels. To allocate and use these resources efficiently, the magnitude of mental health needs and unmet needs in the community is critical information in the decision-making process. The ability to closely monitor and forecast the trends of perceived and unmet needs in populations can greatly facilitate this decision-making process. The objective of this study was to develop prediction models for estimating and forecasting perceived and unmet mental health needs at the provincial and health regional levels in Canada.

## Methods

### Study Design

For the objective of this study, data from the 2018, 2019, and 2020 CCHS and Canadian Urban Environment (CANUE) were used. CCHS is an annual national population health survey conducted by Statistics Canada to gather data on health status, health care use, and health determinants at the health region levels of geography. The CCHS covers the household population from 12 years of age and older living in the 10 provinces and 3 territories. The CCHS sample is selected using different frames according to the age group. For the adult population (18 y and older), the sample of households is selected from an area frame. For the youth population (12 to 17 y old) a list frame is used to select persons. The area frame used by the Labour Force Survey is used as a sampling frame for the adult population. The Labour Force Survey uses a 2-stage sample design. In the first stage, a sample of primary sampling units, corresponding to geographical regions called clusters, is selected. In each selected primary sampling unit, a sample of dwellings is drawn at the second stage. To sample persons for the youth population between the ages of 12 and 17 years, the CCHS uses a list frame created from the Canadian Child Benefit files. The sample for the youth population is selected from a list of individuals. For adult participants, 1 person is selected per household using varying probabilities taking into account the age and the household composition. Each year the CCHS collects information on approximately 65,000 Canadians (60,000 aged 18 y and older and 5000 aged 12 to 17 y) [15]. The CCHS uses 2 separate computer-assisted interviewing applications to collect data, 1 for telephone interviews (computer-assisted telephone interviewing) and 1 for personal interviews (computer-assisted personal interviewing). Approximately 25% of these completed cases were conducted in person using computer-assisted personal interviewing, and the other 75% were conducted over the phone using computer-assisted telephone interviewing. Starting in 2015, the CCHS undertook significant redesign in sampling and the content [15]. Data about the outcome variables of the proposed study (perceived and unmet mental heath needs) were not collected in 2015, 2016, and 2017 CCHS, therefore, we will analyze the 2018, 2019, and 2020 CCHS data.

CANUE is a Canadian Institutes of Health Research–funded collaboration that focuses on developing robust methods for producing measures of urban form that capture a wide range of characteristics for every postal code in Canada [16]. It has produced a unique repository of standardized metrics of urban, suburban, and rural characteristics. CANUE shares a range of data that can be directly linked with population health surveys through postal codes, including neighborhood-level material and social deprivation [17], marginalization [18], and walkability. CANUE is updated every 5 years. The most recent CANUE update was in 2016. The CANUE data can be obtained from CANUE consortium at no costs for research, and is linkable to the CCHS by postal codes.

### Perceived and Unmet Mental Health Needs

In the CCHS, perceived and unmet mental health needs were measured by the Meadows et al [19] Perceived Needs for Care Questionnaire (PNCQ). The PNCQ was designed and field tested for the Australian National Survey for Mental Health and Wellbeing which was commissioned by the Federal Government of Australia [19]. The PNCQ assessed four types of help for problems with emotions, mental health, or the use of alcohol or drugs: (1) information about problems, treatments, or services; (2) medication; (3) counseling or therapy; and (4) other mental health services. Respondents were asked which types of help they had received in the past 12 months. For each type received, they were asked if they felt they had received enough. For each type of help not received, they were asked if they felt it was needed. Based on the PNCQ, the following binary outcome variables can be derived: [9,20]

- *Perceived mental health needs:* having reported “yes” to a perceived need of any type of help.
- *Unmet mental health needs:* For any type of help for which a respondent had a perceived need, having reported not receiving any or enough of that type of help. Interrater reliability of the PNCQ (κ value) was 0.62 [19]. Validity testing using homo-method agreements and hetero-method agreement demonstrated that the PNCQ has good discriminative validity [19]. The PNCQ has been used to assess perceived and unmet mental health needs in a number of population health surveys conducted in Australia [19], Canada [21], the United States [22], and the Netherlands [20].

### Candidate Predictors

#### Overview

Guided by the Andersen Health Behavior model [23], we selected potential predictors from the core component of the CCHS which are consistently administered over time in all provinces and territories. This ensured that health regions can readily ascertain the predictor profile of their regions to make an estimation and prediction when PNCQ is not administered in the CCHS.

From the CCHS core component, we selected the predisposing factors (sex, gender, age, marital status, race or ethnicity, rural or urban residence, first official language spoken, immigration, household food security, self-reported life stress, work stress, life satisfaction, and sense of belonging), enabling factors (educational level, household income, employment status, and insurance coverage), and need factors (general health, comorbid physical conditions, self-reported mental health, mood, and anxiety, and primary care service use). Mental health problems such as depression, anxiety, and psychological distress are not part of the core component; data about these variables are not available in every CCHS and for every province and territory. Therefore, these variables were not selected. A number of studies have shown that health behaviors (smoking [24], problematic substance use [25,26], and physical inactivity [27,28]) are associated with depression, anxiety, and psychological distress. We selected these health behaviors from the core component as candidate predictors. From the CANUE, neighborhood-level social and material deprivation [17], marginalization [18], and walkability will be selected as enabling factors.

#### Model Development

The 2018 CCHS data were used to develop the prediction models. Given the vast geographic area of Canada, we developed the models by regions: Atlantic region (Newfoundland and Labrador, Prince Edward Island, Nova Scotia, and New Brunswick), Central region (Ontario and Quebec), Western region (Manitoba, Saskatchewan, Alberta, and British Columbia). For each region, we developed prediction models for perceived and unmet mental health needs through the application of regression synthetic estimation developed by the UK’s Office of National Statistics [29], which is a type of small area estimation methodology. The same methodology has been used to estimate state and area-level prevalence of severe mental illness in the United States [30]. The regression synthetic estimation method involves several steps: (1) The development of a prediction model of the outcome at the individual level. (2) The coefficients derived from the foregoing model are used with a parallel set of predictors on the area level, using data obtained from CCHS, and coded using the same categories as used to estimate the individual-level model and to compute national and area-level estimates. (3) Internal and external validation.

To develop the predictive models for the outcomes at the health region level, multilevel, random intercept binary logistic regression models were used to analyze the perceived and unmet need for mental health services (level 1) nested within health regions (level 2). A backward selection method was used to identify the model with the best calibration and discrimination. The decisions of model selection were initially based on the changes in the values of Akaike information criterion and Bayesian information criterion [31].

Because the goal of models was to assist decision makers and service planners to estimate perceived and unmet needs at the population level (not to be used by clinicians to identify high risk individuals), we focused more on the calibration of the model, instead of discrimination, when it came to model performance. Discrimination is the ability of a prediction model to separate those who experienced the outcome events from those who did not, by predicting higher versus lower probabilities, respectively. We quantified this by calculating the area under the receiver operating characteristics curve, which is equal to the C statistic. Calibration measures how closely predicted outcomes agree with actual outcomes. For this, we used the calibration slope by which a value of 1 indicates perfect model calibration with data; less than 1 is overconfident and greater than 1 is underconfident [32]. Additionally, we used calibration plots to visually compare the mean predicted risk of the outcomes versus the observed risk (the cumulative fraction of events) by decile risk groups so that the overall calibration and the areas with over or under prediction can be identified.

The second step was synthetic estimation of the proportions at the population level with perceived and unmet mental health needs, which consisted of 2 stages. First, population proportions for predictor variables that were used in the initial modeling were estimated. For instance, if 5 household income categories are used in the initial modeling (<CAD $30,000, CAD $30,000‐$49,000, CAD $49,000‐$60,000, CAD $60,000‐$80,000, and CAD $80,000+; <US $21,175.80, US $21,175.80-$34,587.14, US $34,587.14-$42,351.60, US $42,351.60-56,468.80, and US $56,468.80+, respectively), the population proportion of participants in each of the same 5 income ranges were estimated from the CCHS. The regression coefficients were then applied to the corresponding proportions in the dataset, and to calculate the logit estimates for each province, which were then converted into probabilities, giving the predicted proportions of perceived and unmet mental health needs in the province. These procedures were repeated with data aggregated at the provincial and health regional levels. All analyses were conducted using bootstrap weights provided by Statistics Canada to account for the unequal sampling probabilities and design effects.

#### Validation

As internal validation, we used CCHS-2018 data to compare the predicted proportions (step 2 above) and the observed proportions. To ensure the validity of the predictive models, it is also important that the developed models are externally validated in related but independent populations. As external validation, we applied the developed synthetic models in CCHS-2019 and CCHS-2020, to examine the performance of the models, to investigate how the pandemic may affect the performance of the models by comparing the model predicted (synthetic) proportions and the observed proportions. Additionally, we randomly selected 10 subprovincial health regions from over 100 across the country. We applied the regional models directly to the health regions and compared the observed and predicted proportions of perceived and unmet mental health needs. All analyses were conducted using the bootstrap weights provided by Statistics Canada to account for the sampling and design effects of the CCHS.

### Ethical Considerations

This is a secondary data analysis using the deidentified data collected by Statistics Canada. The data analysis was carried out at one of the Research Data Centers across the country, located on the campus of Dalhousie University. Researchers who conducted data analysis had to go through security clearances and become “deemed employees” of Statistics Canada. The results were vetted by data analysts of the Research Data Center to ensure privacy and confidentiality. CCHS participants gave informed consent to Statistics Canada for use of their data in accordance with the Statistics Act and no compensation was provided to the CCHS participants. For these reasons, ethics review was waived by the health research ethics committee of Dalhousie University.

## Results

### Overview

The 2018 CCHS data were used to develop the prediction models. The demographic and socioeconomic characteristics of the participants in the 2018 CCHS are presented in Table 1. In the 2018 CCHS, 17.82% reported perceived mental health need and 3.81% reported unmet mental health need. In 2019, the proportion of perceived and unmet mental health needs was 18.04% and 3.91%, respectively; in 2020, the proportion of perceived and unmet mental health needs was 18.1% and 3.92%, respectively.

**Table 1.** The demographic and socioeconomic characteristics of the participants in the 2018 cross-sectional Canadian Community Health Survey.

Variables	Values
Men, weighted %	49.4
Women, weighted %	50.6
Age (years), mean (SD)	45.7 (0.14)
Married, common law, or partner, weighted %	58.2
Single, weighted %	30.1
Divorced, separated, or widowed, weighted %	11.7
<High school, weighted %	17.4
High school, weighted %	22.6
≥College or university, weighted %	60
Employed, weighted %	67.2
Unemployed, weighted %	22.4
<15 or >75 years old^a^, weighted %	10.4
Immigrants, weighted %	27.1
Nonimmigrants, weighted %	72.9
White, weighted %	75.5
Non-White, weighted %	24.5
Rural residence, weighted %	17.2
Urban residence, weighted %	82.8
Have insurance for medication, weighted %	19.7
No, weighted %	80.3

a Participants aged <15 or 75+ years were not eligible for the employment question.

### Predicting Perceived Mental Health Need

The individual based models with the best performance for predicting perceived mental health need are in supplemental tables (Tables S1 [Atlantic region], S2 [Central region], and S3 [Western region] in Multimedia Appendix 1). Sex, age, self-reported mental health, physician diagnosed mood and anxiety disorders, and self-reported life stress and life satisfaction were the predictors in the 3 regional models. Perceived work stress and household food insecurity were in the models for the Atlantic and Central regions, but not for the Western region. However, immigrants, smoking, or problematic drinking and material deprivation were predictors in the models for the Atlantic and the Western region, not for the Central region. The individual based models had good discriminative power with C statistics over 0.83 (Tables S4-S6 in Multimedia Appendix 1) and good calibration by comparing observed and predicted proportions.

Converting the individual based models into synthetic estimation models, the predicted proportion of perceived mental health need in each province was estimated. The absolute difference between the observed and predicted proportions in 2018 was less than 1% for all provinces (Tables 2-4), except Saskatchewan (3.37%). This is not surprising as the models were developed using the 2018 CCHS data. To validate the models, we applied the synthetic models directly in 2019 and 2020 data. The extent to which the predicted or synthetic estimates agree with observed proportions varied by regions (Tables 2-4). The models had the best performance in Ontario and British Columbia (absolute difference <1% in both 2019 and 2020), followed by Quebec (absolute difference=1.54% in 2019 and 0.54% in 2020). The models for Nova Scotia, New Brunswick, Saskatchewan, and Alberta had 1 absolute difference being less than 1% and the other greater than 2%. The absolute differences between observed and predicted proportions in Newfoundland and Labrador (−4.16% for perceived needs in 2020) and Prince Edward Island (4.58% for perceived needs in 2019) were larger than those in other provinces. When applying the models in the 10 selected health regions, the data showed that the model performed well in health regions in Ontario (first 2 digits with 35) and in Quebec (first 2 digits with 24), supported by the relatively small absolute differences between observed and predicted proportions (Table 5). Health region 1304 had the largest absolute differences in 2019 and 2020.

**Table 2.** The observed and predicted proportions of perceived and unmet mental health needs in the Atlantic provinces in the 2018, 2019, and 2020 Canadian Community Health Survey.

		Observed proportion (%)	Predicted proportion (%)	Absolute difference^a^ (%)
Newfoundland and Labrador				
Perceived need				
	2018	14.9	14.57	−0.33
	2019	16.24	14.04	−2.2
	2020	19.35	15.19	−4.16
Unmet need				
	2018	2.43	2.07	−0.36
	2019	2.93	1.94	−0.99
	2020	2.43	1.96	−0.47
Prince Edward Island				
Perceived need				
	2018	14.37	14.23	−0.14
	2019	20.92	16.34	−4.58
	2020	16.89	14.93	−1.96
Unmet need				
	2018	3.63	3.96	0.33
	2019	2.74	4.15	1.41
	2020	2.6	3.85	1.25
Nova Scotia				
Perceived need				
	2018	19.81	19.15	−0.66
	2019	23.58	20.44	−3.14
	2020	20.56	21.33	0.77
Unmet need				
	2018	3.43	3.52	0.09
	2019	5.78	3.55	−2.23
	2020	4.23	3.3	−0.93
New Brunswick				
Perceived need				
	2018	17.66	18.44	0.78
	2019	17.22	19.97	2.75
	2020	20	20.83	0.83
Unmet need				
	2018	3.05	2.85	−0.2
	2019	3.85	3.52	−0.33
	2020	3.71	3.42	−0.29

a Absolute difference=predicted proportion–observed proportion.

**Table 3.** The observed and predicted proportions of perceived and unmet mental health needs in Ontario and Quebec in the 2018, 2019, and 2020 Canadian Community Health Survey.

			Observed proportion (%)	Predicted proportion (%)	Absolute difference^a^ (%)
Ontario					
	Perceived need				
		2018	16.93	16.63	−0.30
		2019	17.35	16.94	−0.41
		2020	17.97	17.44	−0.53
	Unmet need				
		2018	3.96	3.98	0.02
		2019	4.08	4.09	0.01
		2020	4	4.29	0.29
Quebec					
	Perceived need				
		2018	17.08	17.65	−0.43
		2019	16.02	17.56	1.54
		2020	16.72	17.26	0.54
	Unmet need				
		2018	3.25	3.26	0.01
		2019	3.01	3.21	0.2
		2020	3.36	3.09	−0.27

a Absolute difference=predicted proportion−observed proportion.

**Table 4.** The observed and predicted proportions of perceived and unmet mental health needs in the Western provinces in the 2018, 2019, and 2020 Canadian Community Health Survey.

			Observed proportion (%)	Predicted proportion (%)	Absolute difference^a^ (%)
Manitoba					
	Perceived need				
		2018	18.8	19.52	0.72
		2019	18.86	19.56	0.7
		2020	16.87	20.13	3.26
	Unmet need				
		2018	3.59	4.18	0.59
		2019	5.11	4.12	−0.99
		2020	4.25	4.43	0.18
Saskatchewan					
	Perceived need				
		2018	16.57	19.94	3.37
		2019	18.61	21.14	2.53
		2020	15.98	21.31	5.33
	Unmet need				
		2018	3.1	4.03	0.93
		2019	2.84	4.23	1.39
		2020	4.07	4.16	0.09
Alberta					
	Perceived need				
		2018	21.73	20.77	−0.96
		2019	20.02	20.91	0.89
		2020	18.92	21.49	2.57
	Unmet need				
		2018	4.52	4.41	−0.11
		2019	3.66	4.43	0.77
		2020	3.72	4.54	0.82
British Columbia					
	Perceived need				
		2018	18.39	18.38	−0.01
		2019	19.14	18.86	−0.28
		2020	20.05	20.01	−0.04
	Unmet need				
		2018	4.28	4.16	−0.12
		2019	4.9	N/A^b^	N/A
		2020	4.87	4.51	−0.36

a Absolute difference=predicted proportion–observed proportion.

b N/A: household food insecurity data were not collected in British Columbia in 2019.

**Table 5.** The observed and predicted proportions of perceived mental health needs by selected health regions in 2018, 2019, and 2020 Canadian Community Health Survey.

Health regions and years		Observed proportion (%)	Predicted proportion (%)	Absolute difference^a^ (%)
1301				
	2018	17.42	19.08	1.66
	2019	19.81	22.47	2.66
	2020	22.89	22.48	−0.41
1304				
	2018	11.86	16.12	4.26
	2019	6.59	15.23	8.64
	2020	9.45	19.61	10.16
2404				
	2018	14.22	16.04	1.82
	2019	14.01	15.83	1.82
	2020	10.78	14.72	3.94
3544				
	2018	20.12	19.59	−0.53
	2019	20.4	18.7	−1.7
	2020	20.13	20.36	0.23
3570				
	2018	12.57	14.91	2.34
	2019	16.29	15.36	−0.93
	2020	17.97	15.87	−2.1
3536				
	2018	14.99	15.9	0.91
	2019	17.4	16.16	−1.24
	2020	17.21	16.38	−0.83
5913				
	2018	15.17	18.28	3.11
	2019	N/A^b^	N/A	N/A
	2020	24.29	18.8	−5.49
5921				
	2018	13.75	18.66	4.91
	2019	N/A	N/A	N/A
	2020	21.24	20.54	−0.7
5922				
	2018	15.8	18.44	2.64
	2019	N/A	N/A	N/A
	2020	19.77	19.46	−0.31
5943				
	2018	17.88	17.71	−0.17
	2019	N/A	N/A	N/A
	2020	23.37	17.84	−5.53

a Absolute difference=predicted proportion–observed proportion.

b N/A: estimates were not available due to lack of data on household food insecurity.

### Predicting Unmet Mental Health Need

The individual based models with the best performance for predicting unmet mental health need are in Tables S7 (Atlantic region), S8 (Central region), and S9 (Western region) in Multimedia Appendix 1. As seen from the tables, sex, age, marital status, self-reported mental health, self-reported life stress, and low sense of belonging to the community were the common predictors in the models for the 3 regions. Household income was a predictor only in the model for Central region; professional diagnosed mood disorders, problematic drinking, and smoking and social deprivation were the factors specific for the model for the Atlantic region. The individual based models had good discriminative power with C statistics over 0.77 (Tables S10-S12 in Multimedia Appendix 1) and good calibration by comparing observed and predicted proportions.

The individual based models were converted into synthetic estimation models, and the predicted proportion of perceived mental health need in each province was estimated (Tables 2-4). The data demonstrated that the models for unmet mental health need performed well in most of the provinces with the absolute difference between observed and predicted proportions being less than 1%, except for Prince Edward Island, Nova Scotia, and Saskatchewan. When applying the models in the 10 selected health regions, the data showed that the models calibrated well with data in all selected health regions with absolute difference between observed and predicted proportions being less than 1% (Table S13 in Multimedia Appendix 1).

## Discussion

### Principal Results

The 3 waves of CCHS showed that the proportion of the Canadian population that perceived the need for mental health care was between 17.82% and 18.1%, and about 4% reported unmet mental health need. This study demonstrated the feasibility of integrating individual and community level data to build informative synthetic models for perceived and unmet mental health care needs at the population level. The models performed well in predicting the outcomes at both the provincial and health regional levels, particularly in populous provinces such as Ontario, Quebec, and British Columbia. The absolute differences between the observed and predicted proportions of perceived and unmet mental health needs in Ontario, Quebec, and British Columbia were less than 1%. For the rest of the provinces, the results showed that the models at provincial and health regional levels predicting unmet mental health needs had better calibration than the models predicting perceived mental health need.

### Limitations

This study had several limitations. First, although the predictors in the models were associated with the outcomes, causal inferences cannot be made. The goal of the prediction models is to identify a key set of factors that in combination are best predictive of the outcomes. The models are not to test hypothesis or make inferences about etiology. Second, the relationships between the selected factors and the outcomes are complex. The logistic regression model is a linear function. Although we found no evidence of interactions among the selected predictors, nonlinear relationships between some predictors and the outcomes are still possible. Future studies may test if models using machine learning techniques have better performance. Third, the selection of candidate predictors was limited by what CCHS collected. It is possible that other factors are associated with the outcomes of this study, but were not measured in CCHS, therefore could not be examined in this study. Finally, the models appeared to perform well at regional and provincial levels. Relatively large absolute differences between predicted and observed proportions of perceived and unmet needs were observed in some health regions (eg, health region 1304). Caution should be exercised for using them at the health region level.

### Comparison With Prior Work

The CCHS used a nationwide representative sample of the Canadian household population. However, we found that a model developed for the whole population performed well only in the most populous provinces such as Ontario and Quebec, but not in the rest of the provinces. Developing models for specific regions appeared to be a better approach. The regional models have some common predictors, including sex, age, self-reported mental health, physician diagnosed mood and anxiety disorders, self-reported life stress and life satisfaction for perceived mental health need, and sex, age, marital status, self-reported mental health, self-reported life stress, and low sense of belonging to the community for unmet mental health need. These factors have been found to be significantly associated with mental health problems [33] in the literature. On the other hand, the coefficients associated with these factors varied by regional models and these models also contained predictors that are regionally specific. This finding indicates that there are common factors in the mechanisms underlying perceived and unmet mental health needs across geographic regions and populations. The extent to which these factors influence perceived and unmet mental health needs may differ due to the population characteristics of these provinces and regions. The differences in population characteristics and the distribution of the outcomes across the regions may also contribute to the finding that the models contained region specific predictors.

We have not found studies on predicting perceived and unmet mental health needs at the population level. Therefore, a direct comparison with previous studies is not possible. There are several studies predicting county or state level suicide and severe mental illness. For example, Kandula et al [34] modelled county-level suicide risk in the United States using county-level predictors derived from 8 different databases of different sources (government programs, health surveys, or private organizations). For some predictors such as the prevalence of major depressive episode, only state-level estimates were available and these estimates were extrapolated to the counties [34]. Hudson [30] explored the utility of regression synthetic estimation model that incorporated individual data from the National Comorbidity Survey, census and hospital administrative data to predict state-level prevalence of severe mental illness. The advantages of these population risk prediction models are the use of community level predictors from existing sources or published research and the ability of adapting the models in local context. Notably, our study used the regression synthetic estimation modeling approach. We used CCHS data. The use of a single data source may improve the efficiency of data analysis, data access, and eventually of the decision-making process.

One critical element of building risk prediction models is assessing model performance and model validation. This is to ensure that the developed model is accurate and has good performance in different populations or at different time periods. In this study, we developed the models using the 2018 CCHS data, and validated the models using the data from 2019 and 2020 CCHS. Furthermore, we validated the models in a random sample of health regions. These models were designed to estimate population proportions and to identify regions with high levels of mental health need and unmet mental health need; these models were not to be used by clinicians to identify high-risk individuals. Therefore, the focus of model performance assessment can be different. Kandula et al [34] used symmetric proportional error (observed deaths–predicted deaths)/(observed deaths+predicted deaths) to quantify model calibration. Hudson [30] calculated the absolute difference between the predicted and observed prevalence of severe mental illness. In this study, we calculated the absolute difference between the observed and model predicted proportions of perceived and unmet mental health needs. The absolute difference indicates the extent to which the model prediction deviates from the observed value. However, there is no consensus about what an optimal threshold of absolute difference should be. There may be other indicators that are useful for assessing the performance of population level models. Consultations with policy and decision makers (ie, end users of the models) would be helpful to understand what indicators are informative about model performance, and the level of the model error that is acceptable.

The results of this study are expected to have implications for population mental health planning. Few would deny that resources allocation should be partly driven by needs, and needs assessments typically require the knowledge of potential changes in prevalence estimates and in local population profiles, for example, their demographics, diagnoses, and mental health services use. The prediction models developed by this study will allow decision makers and mental health services planners to forecast the proportions of perceived and unmet mental health needs in the years to come at the provincial (state) and health regional (county) levels based on the potential changes in local population profiles. Such profile changes may be estimated using health administrative data and national population census data. Additionally, region-specific estimates can help categorize health regions—for example, regions with relatively stable mental health needs especially those that remained in the highest or lowest groups, or regions in which the largest year-to-year changes are observed—and hence help identify areas with a greater need of preventive resources, or conversely identify areas where interventions seem to be effective.

### Conclusion

In conclusion, accurate forecast of perceived and unmet mental health needs in the population can allow policy and decision makers and mental health services planners to categorize regions or communities that are at high need and to monitor changes so that they may mobilize resources and interventions to the right populations and the right places at the right time. Regularly collected population health data such as those from the CCHS are readily accessible to policy and decision makers and mental health services planners. The models are particularly useful for service planners at the health regional level because population health surveys usually do not contain sufficient numbers of participants at the health regional level. Future studies are needed to identify methods to improve prediction for regions with small numbers of residents.

## Supplementary material

10.2196/66056 Multimedia Appendix 1 Supplementary tables.

## Abbreviations

CANUE: Canadian Urban Environment
CCHS: Canadian Community Health Survey
PNCQ: Perceived Needs for Care Questionnaire
