# Supplementary material for: Development and Validation of Prediction Models for Perceived and Unmet Mental Health Needs in the Canadian General Population: Model-Based Synthetic Estimation Study
Source: JMIR Public Health Surveill. 2025 Feb 19;11:e66056. doi: 10.2196/66056 (PMC11864089; doi:10.2196/66056)
Supplement: Multimedia Appendix 1 [file publichealth-v11-e66056-s001.docx]

**Table S1.** The prediction model for perceived mental health needs in the Atlantic region, developed using the data from the 2018 Canadian Community Health Survey.

| **Predictors** | **Coefficients** |
| --- | --- |
| Female | 0.5627 |
| Age | -0.0292 |
| ***Education levels*** | |
| High school education | -0.0920 |
| College, university+ | 0.4328 |
| ***Employment status*** | |
| Unemployed | 0.2309 |
| Not applicable due to age (<15, >75 years) | 0.3525 |
| Immigrants | -0.5323 |
| Self-reported mental health  (good, fair, poor) | 0.9314 |
| Mood disorders diagnosed by health professional | 2.0877 |
| Anxiety disorders diagnosed by health professional | 1.6919 |
| Self-reported life stress | 0.6008 |
| Self-reported work stress | 0.3335 |
| ***Life satisfaction*** | |
| Satisfied | 0.3448 |
| Neither – very dissatisfied | 0.4598 |
| ***Household food insecurity*** | |
| Marginally secure | 0.6717 |
| Moderately insecure | 0.0352 |
| Very insecure | -0.2470 |
| Smoking | -0.2520 |
| Material deprivation | -3.2637 |
| ***Provinces*** | |
| Prince Edward Island | -0.1031 |
| Nova Scotia | 0.2847 |
| New Brunswick | 0.3559 |
| Newfoundland and Labrador | 0 |
|  |  |

**Table S2.** The prediction model for perceived mental health needs in Central region (Ontario and Quebec), developed using the data from the 2018 Canadian Community Health Survey.

| **Predictors** | **Coefficients** |
| --- | --- |
| Female | 0.3814 |
| Age | -0.0228 |
| Immigrants | -0.4846 |
| Urban residence | 0.1688 |
| Self-reported mental health  (good, fair, poor) | 0.8013 |
| Mood disorders diagnosed by health professional | 1.9269 |
| Anxiety disorders diagnosed by health professional | 1.3786 |
| Self-reported life stress | 0.4842 |
| Self-reported work stress | 0.2979 |
| ***Life satisfaction*** | |
| Satisfied | 0.6142 |
| Neither – very dissatisfied | 0.8428 |
| ***Household food insecurity*** | |
| Marginally secure | 0.2318 |
| Moderately insecure | 0.5359 |
| Very insecure | 1.0473 |
| ***Province*** | |
| Ontario | -0.1717 |

**Table S3.** The prediction model for perceived mental health needs in the Western Canada (Manitoba, Saskatchewan, Alberta, British Columbia), developed using the data from the 2018 Canadian Community Health Survey.

| **Predictors** | **Coefficients** |
| --- | --- |
| Female | 0.4797 |
| Age | -0.0344 |
| ***Marital status*** | |
| Single | -0.0437 |
| Divorced/separated/widowed | 0.4225 |
| ***Educational levels*** | |
| High school, college | 0.2802 |
| Less than high school | 0.5438 |
|  | |
| Immigrants | -0.5094 |
| Self-reported mental health  (good, fair, poor) | 0.8525 |
| Self-reported life stress | 0. 5189 |
| Mood disorders diagnosed by health professional | 1.9943 |
| Anxiety disorders diagnosed by health professional | 1.190743 |
| ***Life satisfaction*** | |
| Satisfied | 0.3721 |
| Neither – very dissatisfied | 0.9417 |
|  |  |
| Problem drinking | 0.2976 |
| Material deprivation | -3.1543 |

**Table S4.** The performance of the prediction model for perceived mental health needs in the Atlantic region using the data from the 2018, 2019 and 2020 Canadian Community Health Survey.

|  | 2018* | | 2019 | | 2020 | |
| --- | --- | --- | --- | --- | --- | --- |
|  | Observed proportion | Predicted proportion | Observed proportion | Predicted proportion | Observed proportion | Predicted proportion |
| Overall | 17.70% | 17.59% | 19.79% | 20.65% | 19.89% | 21.81% |
| NF | 14.90% | 14.67% | 16.24% | 15.39% | 19.35% | 17.31% |
| PEI | 14.37% | 14.26% | 20.92% | 19.55% | 16.89% | 17.28% |
| NS | 19.81% | 19.73% | 23.58% | 22.44% | 20.56% | 24.16% |
| NB | 17.66% | 17.56% | 17.22% | 22.27% | 20.00% | 22.98% |
|  | | | | | | |
| AUC | 0.857 | | 0.858 | | 0.862 | |
| Calibration slope | 0.922 | | 0.989 | | 0.981 | |

*: Data in the 2018 CCHS were used to develop the model. The model was validated in 2019 and 2020 data.

NF: Newfoundland and Labrador. PEI: Prince Edward Island. NS: Nova Scotia. NB: New Brunswick.

AUC: Area under ROC curve.

**Table S5.** The performance of the prediction model for perceived mental health needs in the Central region (Ontario and Quebec) using the data from the 2018, 2019 and 2020 Canadian Community Health Survey.

|  | 2018* | | 2019 | | 2020 | |
| --- | --- | --- | --- | --- | --- | --- |
|  | Observed proportion | Predicted proportion | Observed proportion | Predicted proportion | Observed proportion | Predicted proportion |
| Overall | 16.99% | 16.94% | 17.20% | 17.14% | 17.51% | 17.62% |
| Ontario | 16.93% | 16.92% | 17.35% | 17.30% | 17.97% | 17.92% |
| Quebec | 17.08% | 16.98% | 16.02% | 16.88% | 16.72% | 17.13% |
|  | | | | | | |
| ROC | 0.837 | | 0.841 | | 0.840 | |
| Calibration slope | 0.981 | | 0.962 | | 0.996 | |

*: Data in the 2018 CCHS were used to develop the model. The model was validated in 2019 and 2020 data.

AUC: Area under ROC curve.

**Table S6.** The performance of the prediction model for perceived mental health needs in the Western region using the data from the 2018, 2019 and 2020 Canadian Community Health Survey.

|  | 2018* | | 2019 | | 2020 | |
| --- | --- | --- | --- | --- | --- | --- |
|  | Observed proportion | Predicted proportion | Observed proportion | Predicted proportion | Observed proportion | Predicted proportion |
| Overall | 19.51% | 19.75% | 19.38% | 20.30% | 18.91% | 20.55% |
| MB | 18.80% | 20.36% | 18.86% | 20.51% | 16.87% | 20.30% |
| SK | 16.57% | 20.03% | 18.61% | 21.86% | 15.98% | 21.26% |
| AB | 21.73% | 20.80% | 20.02% | 21.05% | 18.92% | 21.05% |
| BC | 18.39% | 18.70% | 19.14% | 19.23% | 20.05% | 20.05% |
|  | | | | | | |
| AUC | 0.831 | | 0.845 | | 0.850 | |
| Calibration slope | 0.958 | | 0.976 | | 0.896 | |

*: Data in the 2018 CCHS were used to develop the model. The model was validated in 2019 and 2020 data.

MB: Manitoba SK: Saskatchewan AB: Alberta BC: British Columbia

AUC: Area under ROC curve.

**Table S7.** The prediction model for unmet mental health needs in the Atlantic region using the data from the 2018 Canadian Community Health Survey.

| **Predictors** | **Coefficients** |
| --- | --- |
| Female | 0.5667 |
| Age | -0.0073 |
| ***Marital status*** | |
| Single | 0.4780 |
| Divorced/separated/widowed | -0.0998 |
| ***Employment status*** | |
| Unemployed | -0.6191 |
| Not applicable due to age (<15, >75 years) | -0.2853 |
|  |  |
| Self-reported mental health  (good, fair, poor) | 0.6801 |
| Mood disorders diagnosed by health professional | 0.3311 |
| Problematic drinking | 0.4803 |
| Self-reported life stress | 1.5643 |
| Self-reported work stress | 0.0147 |
| Low sense of belonging | 0.4443 |
| Smoking | 0.1694 |
| Social deprivation | 3.0151 |
| ***Household food insecurity*** | |
| Marginally secure | 0.2318 |
| Moderately insecure | 0.5359 |
| Very insecure | 1.0473 |
| ***Provinces*** | |
| Prince Edward Island | 0.6696 |
| Nova Scotia | 0.4742 |
| New Brunswick | 0.5085 |
| Newfoundland and Labrador | 0 |
|  |  |

**Table S8.** The prediction model for unmet mental health needs in the Central region (Ontario and Quebec) using the data from the 2018 Canadian Community Health Survey.

| **Predictors** | **Coefficients** |
| --- | --- |
| Female | 0.2851 |
| Age | -0.0161 |
| ***Marital status*** | |
| Single | 0.2161 |
| Divorced/separated/widowed | 0.0984 |
| ***Educational levels*** | |
| High school, college | 0.6112 |
| Less than high school | 0.6596 |
| ***Household income levels*** | |
| $15000-$29999 | -0.1805 |
| $30000 - $89999 | -0.0394 |
| $90000+ | -0.2116 |
| ***Employment status*** | |
| Unemployed | 0. 0847 |
| Not applicable due to age (<15, >75 years) | -0.6618 |
| Self-reported mental health  (good, fair, poor) | 0.8862 |
| Self-reported life stress | 0.8902 |
| Low sense of belonging | 0.4256 |
| ***Life satisfaction*** | |
| Satisfied | 1.1173 |
| Neither – very dissatisfied | 1.2288 |
| ***Province*** | |
| Ontario | 0.2170 |

**Table S9.** The prediction model for unmet mental health needs in the Western Canada (Manitoba, Saskatchewan, Alberta, British Columbia) using the data from the 2018 Canadian Community Health Survey.

| **Predictors** | **Coefficients** |
| --- | --- |
| Female | 0. 1426 |
| Age | -0.0237 |
| ***Marital status*** | |
| Single | -0.0557 |
| Divorced/separated/widowed | 0.3411 |
| ***Educational levels*** | |
| High school, college | 0.6717 |
| Less than high school | 0.8541 |
|  | |
| Self-reported mental health  (good, fair, poor) | 0.8098 |
| Self-reported life stress | 0.4548 |
| High work stress | 0.3384 |
| Low sense of belonging | 0.2483 |
| ***Life satisfaction*** | |
| Satisfied | 0.5651 |
| Neither – very dissatisfied | 1.2330 |
| ***Household food insecurity*** | |
| Marginally secure | 0.4267 |
| Moderately insecure | 0.5343 |
| Very insecure | 0.1736 |

**Table S10.** The performance of the prediction model for unmet mental health needs in Atlantic region using the data from 2018, 2019 and 2020 Canadian Community Health Survey.

|  | 2018* | | 2019 | | 2020 | |
| --- | --- | --- | --- | --- | --- | --- |
|  | Observed proportion | Predicted proportion | Observed proportion | Predicted proportion | Observed proportion | Predicted proportion |
| Overall | 3.10% | 2.86% | 4.35% | 4.71% | 3.57% | 3.70% |
| NF | 2.43% | 1.67% | 2.93% | 2.85% | 2.43% | 2.23% |
| PEI | 3.63% | 3.74% | 2.74% | 6.23% | 2.60% | 4.95% |
| NS | 3.43% | 3.31% | 5.78% | 5.24% | 4.23% | 4.01% |
| NB | 3.05% | 2.96% | 3.85% | 5.01% | 3.71% | 4.07% |
|  | | | | | | |
| AUC | 0.770 | | 0.774 | | 0.804 | |
| Calibration slope | 0.797 | | 0.894 | | 0.964 | |

*: Data in the 2018 CCHS were used to develop the model. The model was validated in 2019 and 2020 data.

NF: Newfoundland and Labrador. PEI: Prince Edward Island. NS: Nova Scotia. NB: New Brunswick.

AUC: Area under ROC curve.

**Table S11.** The performance of the prediction model for unmet mental health needs in Ontario and Quebec using the data from 2018, 2019 and 2020 Canadian Community Health Survey.

|  | 2018* | | 2019 | | 2020 | |
| --- | --- | --- | --- | --- | --- | --- |
|  | Observed proportion | Predicted proportion | Observed proportion | Predicted proportion | Observed proportion | Predicted proportion |
| Overall | 3.70% | 3.75% | 3.69% | 3.87% | 3.76% | 3.88% |
| Quebec | 3.25% | 3.22% | 3.01% | 3.24% | 3.36% | 3.05% |
| Ontario | 3.96% | 4.07% | 4.08% | 4.25% | 4.00% | 4.37% |
|  | | | | | | |
| AUC | 0.785 | | 0.770 | | 0.774 | |
| Calibration slope | 0.965 | | 0.878 | | 0.876 | |

*: Data in the 2018 CCHS were used to develop the model. The model was validated in 2019 and 2020 data.

AUC: Area under ROC curve.

**Table S12.** The performance of the prediction model for unmet mental health needs in the Western region using the data from 2018, 2019 and 2020 Canadian Community Health Survey.

|  | 2018* | | 2019 | | 2020 | |
| --- | --- | --- | --- | --- | --- | --- |
|  | Observed proportion | Predicted proportion | Observed proportion | Predicted proportion | Observed proportion | Predicted proportion |
| Overall | 4.18% | 4.18% | 4.27% | 3.94% | 4.31% | 4.13% |
| MB | 3.59% | 4.18% | 5.11% | 3.85% | 4.25% | 4.27% |
| SK | 3.10% | 3.95% | 2.84% | 3.92% | 4.07% | 3.70% |
| AB | 4.52% | 4.23% | 3.66% | 3.98% | 3.72% | 4.16% |
| BC | 4.28% | 4.19% | 4.90% | NA | 4.87% | 4.18% |
|  | | | | | | |
| AUC | 0.763 | | 0.782 | | 0.794 | |
| Calibration slope | 0.976 | | 0.957 | | 1.04 | |

*: Data in the 2018 CCHS were used to develop the model. The model was validated in 2019 and 2020 data.

MB: Manitoba SK: Saskatchewan AB: Alberta BC: British Columbia

AUC: Area under ROC curve.

NA: Not available as food insecurity was not available in BC.

**Table S13.** The observed and predicted proportions of unmet mental health needs by the western provinces using the data from 2018, 2019 and 2020 Canadian Community Health Survey.

| **Health regions** | **Years** | **Observed proportion** | **Predicted proportion** | **Absolute difference** |
| --- | --- | --- | --- | --- |
| ***Unmet mental health needs*** | | | | |
| 2404 | 2018 | 2.84% | 2.76% | -0.08% |
|  | 2019 | 2.61% | 2.68% | 0.07% |
|  | 2020 |  |  |  |
| 3570 | 2018 | 5.10% | 4.17% | -0.93% |
|  | 2019 | 5.06% | 4.34% | -0.72% |
|  | 2020 | 4.95% | 4.33% | -0.62% |
| 5913 | 2018 | 3.80% | 3.76% | -0.04% |
|  | 2019 |  |  |  |
|  | 2020 |  |  |  |
| 5921 | 2018 | 3.34% | 3.97% | 0.63% |
|  | 2019 |  |  |  |
|  | 2020 |  |  |  |
| 5922 | 2018 | 3.85% | 4.50% | 0.65% |
|  | 2019 |  |  |  |
|  | 2020 |  |  |  |
| 1301 | 2018 |  |  |  |
|  | 2019 | 4.20% | 3.71% | -0.49% |
|  | 2020 |  |  |  |
| 3536 | 2018 |  |  |  |
|  | 2019 | 4.51% | 3.74% | -0.77% |
|  | 2020 | 4.22% | 4.06% | -0.16% |

Blank: small cell size, not releasable.
